# Impact of cystic fibrosis on birthweight: a population based study of children in Denmark and Wales

**DOI:** 10.1136/thoraxjnl-2018-211706

**Published:** 2018-07-19

**Authors:** Daniela K Schlüter, Rowena Griffiths, Abdulfatah Adam, Ashley Akbari, Martin L Heaven, Shantini Paranjothy, Anne-Marie Nybo Andersen, Siobhán B Carr, Tania Pressler, Peter J Diggle, David Taylor-Robinson

**Affiliations:** 1 Centre for Health Informatics, Computing and Statistics (CHICAS), Lancaster Medical School, Lancaster University, Lancaster, UK; 2 Farr Institute, Swansea University Medical School, Swansea University, Swansea, UK; 3 Section of Epidemiology, Department of Public Health, University of Copenhagen, Copenhagen, Denmark; 4 Division of Population Medicine, School of Medicine, Cardiff University, Cardiff, UK; 5 Department of Respiratory Paediatrics, Royal Brompton Hospital, London, UK; 6 Copenhagen Cystic Fibrosis Centre, Department of Infectious Diseases, Rigshospitalet, University of Copenhagen, Copenhagen, Denmark; 7 Department of Public Health and Policy, Farr Institute, University of Liverpool, Liverpool, UK

**Keywords:** cystic fibrosis, clinical epidemiology

## Abstract

**Background:**

Poor growth during infancy and childhood is a characteristic feature of cystic fibrosis (CF). However, the impact of CF on intrauterine growth is unclear. We studied the effect of CF on birth weight in Denmark and Wales, and assessed whether any associations are due to differences in gestational age at birth.

**Methods:**

We conducted national registry linkage studies in two countries, using data for 2.2 million singletons born in Denmark (between 1980 and 2010) and Wales (between 1998 and 2015). We used hospital inpatient and outpatient data to identify 852 children with CF. Using causal mediation methods, we estimated the direct and indirect (via gestational age) effect of CF on birth weight after adjustment for sex, parity and socioeconomic background. We tested the robustness of our results by adjusting for additional factors such as maternal smoking during pregnancy in subpopulations where these data were available.

**Results:**

Babies with CF were more likely to be born preterm and with low birth weight than babies with no CF (12.7% vs 5% and 9.4% vs 5.8% preterm; 11.9% vs 4.2% and 11% vs 5.4% low birth weight in Denmark and Wales, respectively). Using causal mediation methods, the total effect of CF on birth weight was estimated to be −178.8 g (95% CI −225.43 to −134.47 g) in the Danish population and −210.08 g (95% CI −281.97 to −141.5 g) in the Welsh population. About 40% of this effect of CF on birth weight was mediated through gestational age.

**Conclusions:**

CF significantly impacts on intrauterine growth and leads to lower birth weight in babies with CF, which is only partially explained by shorter gestation.

**Key messages**

**What is the key question?**

- Does having cystic fibrosis (CF) impact on birth weight and gestational age at birth?

**What is the bottom line?**

- CF has a large effect on birth weight, similar to the impact of maternal smoking during pregnancy, and is only partially explained by shorter gestation, indicating that having CF impacts directly on intrauterine growth.

**Why read on?**

- Using record-linked data for 2.2 million babies in two countries and modern methods of causal mediation analysis, we show that babies with CF are born about 200 g lighter than babies with no CF, and only around 40% of this effect is explained by reduced gestational age.

## Introduction

Cystic fibrosis (CF) impacts negatively on the growth of children. Suboptimal growth is associated with worse lung function and increased risk of premature death in people with CF.^1–5^ Around the time of diagnosis, children with CF are on average lighter than non CF children^6^ and a number of small studies have suggested that babies with CF may be born lighter than the non-CF population.^1 7–14^ A higher prevalence of preterm birth in babies with CF has been suggested as a possible reason,^7 13^ whereas other studies have suggested that even in babies born at term, those with CF are lighter than those without CF^1^ raising the possibility of prenatal and genetic influences on growth. A limitation of these studies has been that they have not been population-based, and have only captured small samples of children with CF from specific regions or seen at individual care centres.

In children with CF, as in the general population, those from socially disadvantaged backgrounds are smaller and lighter compared with children from more affluent families, and have worse health outcomes and survival. In children with CF these associations are evident at diagnosis, and they appear to track forward influencing subsequent outcomes.^15^ The socioeconomic differences in early growth in children with CF may be a consequence of social differences in birth weight in children with CF, as well as differential weight loss by socioeconomic status in children with CF prior to diagnosis in those that are not diagnosed by new-born screening.

In this study, we aimed to clarify the association of CF with preterm birth and birth weight, and establish whether these associations vary by childhood socioeconomic conditions (SECs), at population level in Denmark and Wales. We further aimed to assess whether any differences in birth weight observed were due to differences in gestational age between babies with CF and babies with no CF, or whether there are separate biological processes linked to CF that lead to reduced birth weight.

## Methods

### Study design, setting, data sources and participants

We undertook a population-level linkage study of all singleton children born in Denmark between 1980 and 2010 and in Wales between 1998 and 2015.

In Denmark, we accessed the data from the Danish Medical Birth Register^16^ linked to the Danish National Patient Register (DNPR)^17^ and to data on highest obtained educational level in Statistics Denmark for all children and their parents. Linkage was based on the Central Person Registry Number.

In Wales, we analysed data from the Secure Anonymised Information Linkage databank.^18 19^ We accessed the National Community of Child Health (NCCH) Database and used Anonymised Linkage Fields to link to the Congenital Anomaly Register and Information Service (CARIS), Patient Episode Database for Wales (PEDW), Welsh Longitudinal General Practice data (WLGP), the Annual District Birth Extracts (ADBE—also known as the Office of National Statistics birth register) and the Welsh Demographics Service Dataset (WDSD).

Multiple births, individuals with missing birth weight, gestational age, parity, sex or deprivation score were excluded from the analysis. We also excluded individuals with birth weight <100 g or >7 kg or gestational age <21 weeks or >45 weeks, on the basis that these may be data entry errors. See  online supplementary material section S.1 for more details.

10.1136/thoraxjnl-2018-211706.supp1 Supplementary data

### Outcome, exposure and covariates

**Figure 1:**
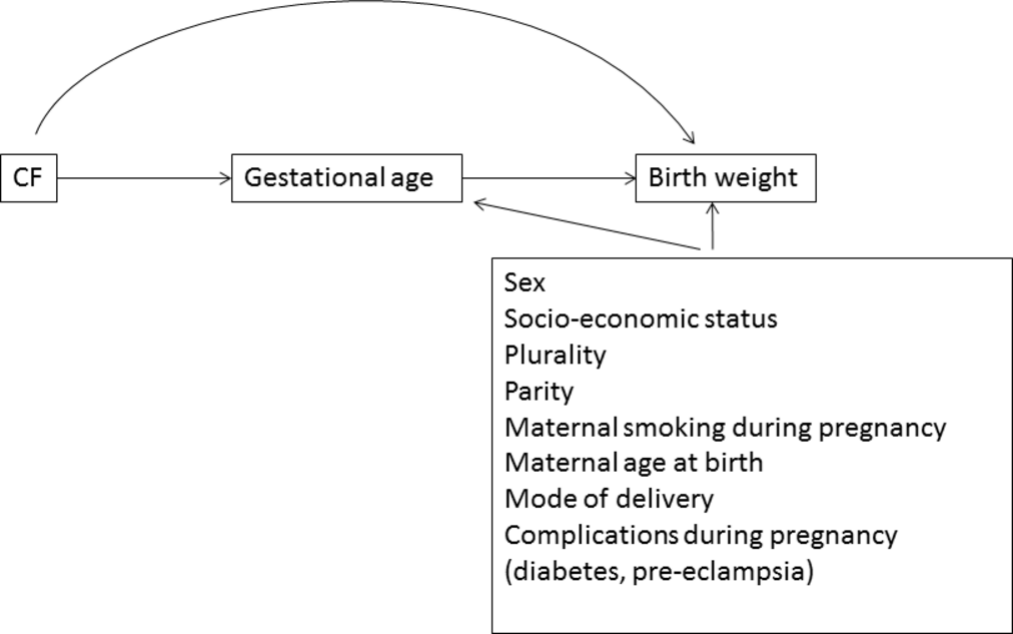
Directed acyclic graph depicting the hypothesised causal framework.

The main outcomes of interest were birth weight and gestational age; and the main exposure of interest was being coded as having CF compared with not having CF in the linked administrative datasets in each country. Selection of covariates in the analysis was informed a priori using a directed acyclic graph (figure 1), and this was used to inform similar, but separate, analyses in Denmark and Wales based on data availability. Further information on the datasets and data cleaning are provided in the online supplementary material section S.1.

#### Danish analysis

In Denmark, CF diagnosis was extracted from the DNPR, which includes hospital inpatient and outpatient data, identified by the  International Classification of Diseases (ICD) 8 code 273 in years prior to 1994 and ICD 10 code E84 thereafter. Birth weight, gestational age, date of birth, sex, parity (from 1996 onwards), mode of delivery, maternal age at birth and maternal smoking (from 1996 onwards) during pregnancy were obtained from the Danish Medical Birth Register. A binary variable indicating first-born status was derived from the available parity variable post-1996; parity for births prior to 1996 was estimated as the number of previous live births and stillbirths, which reached gestational age >22 weeks. Additional available covariates in Denmark were pre-eclampsia or eclampsia during index pregnancy (ICD 8 code 637, ICD 10 code O14 and O15), and diabetes during index pregnancy (ICD 8 code 250, ICD 10 code O24), which were obtained from the DNPR. Highest maternal educational level at birth (grouped as International Standard Classification of Education (ISCED) levels 1–6 and obtained from the Education Register) was used as a measure of childhood SECs at birth.

#### Welsh analysis

In Wales, we obtained information on diagnosis of CF from CARIS, PEDW and WLGP, identified by ICD 10 code E84 and READ codes 1264., 66 k., 66k0., 9No7., C370. We obtained birth weight, week of birth of the baby and sex from the ADBE. Other covariates in the analysis included a binary variable indicating first-born status (derived from the number of previous live births given in NCCH); information on the mother’s smoking status in the year previous to birth and the mother’s week of birth (through WLGP). The mother’s age at birth was calculated as difference in days between week of birth of the baby and week of birth of mother divided by 365.25. Deprivation quintiles of small area of residence based on the Welsh Index of Multiple Deprivation (WIMD) was obtained for the Lower Super Output Area (LSOA) of the mother’s postcode from the WDSD and used as a measure of SECs at birth. The LSOAs from the 2001 census were used together with the WIMD scores from 2008.

### Statistical analysis

We used causal mediation methods^20^ to estimate the direct effect of CF on birth weight after adjustment for gestational age, and the indirect effect of CF on birth weight due to any effect on gestational age, which in turn influences birth weight (figure 1).

We modelled the joint distribution of birth weight and gestational age using a two-component mixture of bivariate linear regression models for the two countries separately. Finite mixture models have previously been used for modelling of gestational age and birth weight in order to capture the skewed shape of their distributions.^21 22^ For parameter estimation, we factorised the joint distribution into the distribution of gestational age multiplied by the distribution of birth weight conditional on gestational age.

We initially included CF, sex, parity and socioeconomic status as explanatory variables for both gestational age, and birth weight conditional on gestational age. The significance of each of the covariates was tested by backward elimination based on the likelihood ratio criterion with a cut-off for significance of p<0.05. The direct effects of CF on birth weight and gestational age after adjustment for possible confounders were estimated in both subpopulations. The indirect effect of CF on birth weight through gestational age was estimated by the product of the effect of CF on gestational age and the effect of gestational age on birth weight.^23^ In the context of linear regression models for both the outcome and the mediator variable, as is the case here, this estimator has been shown to be identical to the average causal mediation effect or natural indirect effect if identifiability assumptions hold^24 25^ (see online supplementary material section S.6 for the assumptions). We estimated the total effects of CF on birth weight in both subpopulations as the sum of the direct and indirect effects.^24^ Effects of CF on birth weight and gestational age at a whole population level were estimated as a weighted average of the effects in the subpopulations; 95% CIs for all effect sizes were estimated by non-parametric bootstrap based on 999 samples. We carried out the analysis in R V.3.3.1 using the package FlexMix^26^ for fitting the models.

### Robustness tests and additional analyses

We performed multiple sensitivity analyses to assess the robustness of our results. We allowed for interaction terms between CF and any of the other covariates and assessed their significance using the likelihood ratio test with cut-off for significance of p<0.05. We repeated the analyses: allowing for an interaction term between CF and gestational age; and including just one child per mother to remove any biases that could be introduced due to correlations between outcomes in children from the same mother. In order to assess the influence of unmeasured confounders in our main analysis, we repeated our analyses in subpopulations in which we have additional data on age of the mother at birth and smoking status in the year prior to birth; in Denmark, we additionally included data on mode of delivery, pre-eclampsia or eclampsia and diabetes during pregnancy. In order to assess the impact of potential misclassification of CF cases, we repeated the analyses using stricter classification criteria for CF. In Denmark, we classified those as CF that had a CF code in the DNPR and that had been seen at a hospital more than once. In Wales, we selected only those individuals that were coded as CF in CARIS. In an additional post hoc analysis using our final model, we estimated the probability for a baby with CF compared with a baby with no CF to be born with low birth weight, since this may also be of clinical interest. See online supplementary material section S.8 for details.

### Ethics and information governance

We use anonymised data in this study, therefore specific ethics approval was not needed.

## Results

### Population characteristics

**Figure 2:**
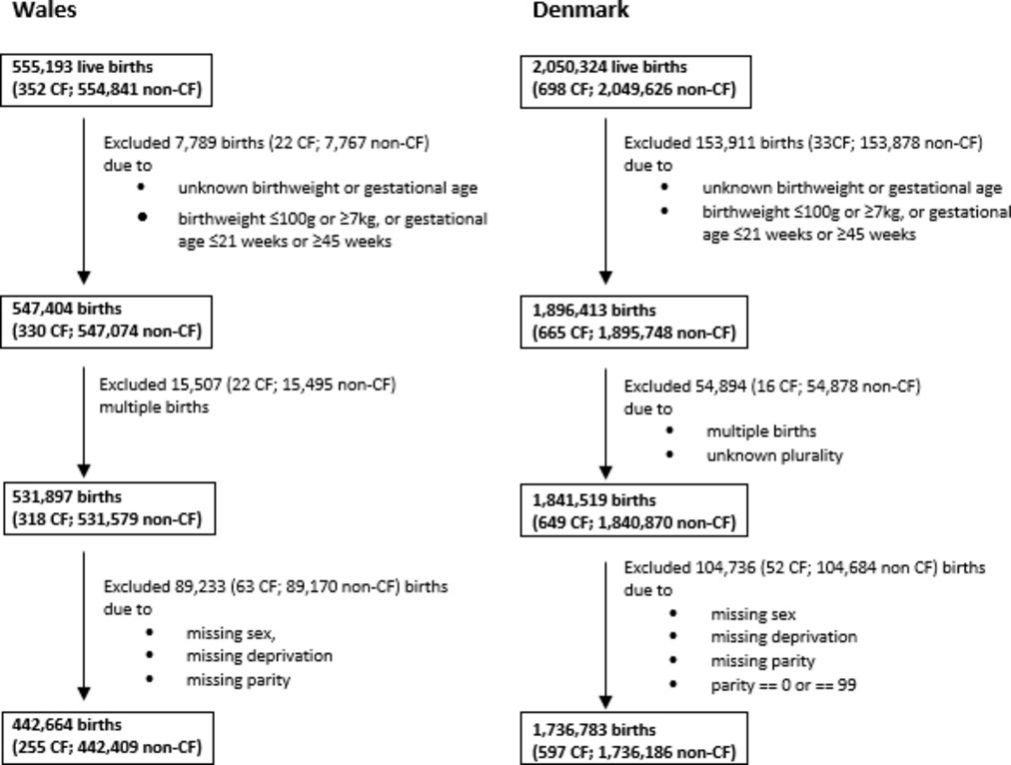
Flow chart showing the derivation of the study population. CF, cystic fibrosis.

Figure 2 shows the derivation of the final study populations. In Denmark and Wales, 597 out of 1 736 186 and 255 out of 442 409 children had CF codes, respectively. The baseline characteristics of both study populations are given in table 1.

**Table 1:** Demographics of the study population

	Wales		Denmark	
	Non-CF population	CF population	Non-CF population	CF population
N	442 409	255	1 736 186	597
Birth weight in g (median (IQR))	3400.00 (3060.00, 3740.00)	3200.00 (2845.00, 3567.50)	3500.00 (3150.00, 3850.00)	3300.00 (2900.00, 3700.00)
Low birth weight=yes (%)	24 035 (5.4)	28 (11.0)	72 480 (4.2)	71 (11.9)
Gestational age in weeks (median (IQR))	40.00 (39.00, 41.00)	39.00 (38.00, 40.00)	40.00 (39.00, 41.00)	39.71 (38.00, 40.43)
Preterm=yes (%)	25 770 (5.8)	24 (9.4)	87 317 (5.0)	76 (12.7)
Sex=male (%)	215 238 (48.7)	116 (45.5)	891 336 (51.3)	320 (53.6)
First-born=yes (%)	205 805 (46.5)	122 (47.8)	523 457 (30.1)	179 (29.8)
Mother’s age at birth (median (IQR))	27.94 (23.67, 32.35)	27.06 (23.07, 31.78)	29.00 (25.00, 32.00)	28.00 (24.00, 31.00)
Mother smoked year prior pregnancy				
No (%)	79 208 (17.9)	48 (18.8)	911 436 (52.5)	239 (40.0)
Yes (%)	42 449 (9.6)	20 (7.8)	286 993 (16.5)	72 (12.1)
NA (%)	320 752 (72.5)	187 (73.3)	537 757 (31.0)	286 (47.9)
WIMD quintile*				
1 (%)	109 986 (24.9)	68 (26.7)	NA	NA
2 (%)	98 595 (22.3)	58 (22.7)	NA	NA
3 (%)	87 144 (19.7)	36 (14.1)	NA	NA
4 (%)	76 076 (17.2)	48 (18.8)	NA	NA
5 (%)	70 608 (16.0)	45 (17.6)	NA	NA
Maternal education†				
Primary or lower secondary (%)	NA	NA	490 215 (28.2)	218 (36.5)
Upper secondary or postsecondary non-tertiary (%)	NA	NA	733 037 (42.2)	233 (39.0)
Tertiary education or not known (%)	NA	NA	512 934 (29.5)	146 (24.5)
Diabetes=no (%)	NA	NA	1 734 670 (99.9)	590 (98.8)
Pre-eclampsia=no (%)	NA	NA	1 733 619 (99.9)	‡
Mode of delivery				
Vaginal (%)	NA	NA	1 249 816 (72.0)	402 (67.3)
Elective caesarean section (%)	NA	NA	79 127 (4.6)	21 (3.5)
Emergency caesarean section (%)	NA	NA	83 866 (4.8)	21 (3.5)
NA (%)	NA	NA	323 377 (18.6)	153 (25.6)

*WIMD, where 1=most deprived and 5=least deprived.

†Original levels are grouped together as follows: ‘primary education, first stage of basic education’ and ‘lower secondary education, second stage of basic education’ are grouped into ‘primary or lower secondary education’; ‘upper secondary education’ and ‘postsecondary, non-tertiary education’ are grouped into ‘upper secondary or postsecondary non-tertiary’; ‘first stage of tertiary education’, ‘second stage of tertiary education’ and ‘not known’ are grouped into ‘tertiary education or not known’.

‡Omitted due to cells containing five or fewer individuals or because it would allow the calculation of a cell with five or fewer individuals.

CF, cystic fibrosis; NA, not available; WIMD, Welsh Index of Multiple Deprivation.

In Denmark, 11.9% of the CF population had low birth weight (<2.5 kg) compared with 4.2% in the non-CF population; in Wales, 11% compared with 5.4% had low birth weight. In Denmark, 12.7% of the CF-coded babies were preterm (<37 weeks) compared with 5% in the non-CF population. In Wales, 9.4% of the CF-coded babies were preterm compared with 5.8% in the non-CF population. Socioeconomic position, first-born status, gender, age of the mother at birth, smoking in the year prior to birth, mode of delivery and rates of diabetes and pre-eclampsia were distributed similarly in the CF and non-CF-coded populations.

### The effect of cystic fibrosis on birth weight and gestational age

The estimated whole population effects are discussed in this section; for details on the mixture subpopulations and the estimated effects at the level of the subpopulations, see online supplementary material section S.2 and S.4, respectively. All explanatory variables included in the model were significant at the 5% level (see online supplementary material section S.3). Tables 2 and 3 give the estimated effects of the explanatory factors on gestational age and birth weight, respectively.

**Table 2:** Estimated effects on gestational age in weeks

	Wales	Denmark
CF (95% CI)	−0.57 (−0.83 to –0.33)	−0.41 (−0.55 to –0.25)
First-born=yes (95% CI)	0.16 (0.15 to 0.17)	0.03 (0.02 to 0.03)
Sex=male (95% CI)	−0.06 (−0.07 to –0.05)	−0.04 (−0.05 to –0.04)
WIMD (95% CI)*		
1	−0.11 (−0.13 to –0.09)	NA
2	−0.05 (−0.07 to –0.03)	NA
3	−0.02 (−0.04 to 0)	NA
4	0.01 (−0.01 to 0.03)	NA
Maternal education (95% CI)†		
Lower secondary education, second stage of basic education	NA	0.01 (−0.02 to 0.05)
Upper secondary education	NA	0.15 (0.12 to 0.18)
Postsecondary non-tertiary education	NA	0.28 (0.09 to 0.46)
First stage of tertiary education, not advanced research qualification	NA	0.23 (0.2 to 0.26)
Second stage of tertiary education, advanced research qualification	NA	0.28 (0.21 to 0.35)
Not known	NA	0.23 (0.15 to 0.32)

*WIMD, where 1=most deprived and 5=least deprived, reference level WIMD=5.

†Reference level Maternal education= Primary education, first stage of basic education.

CF, cystic fibrosis; NA, not available; WIMD, Welsh Index of Multiple Deprivation.

**Table 3:** Estimated effects on birth weight in grams

	Wales	Denmark
Gestational age (95% CI) (g per week)	139.58 (137.86 to 141.21)	141.42 (140.85 to 142.2)
CF direct (95% CI)	−127.24 (−182 to –72.61)	−116.09 (−152.53 to –80.5)
CF indirect (95% CI)	−82.85 (−122.22 to –44.91)	−62.71 (−84.47 to –39.96)
CF total (95% CI)	−210.08 (−281.97 to –141.5)	−178.8 (−225.43 to –134.47)
First-born=yes (95% CI)	−115.21 (−117.84 to –112.54)	−113.57 (−115.38 to –112.45)
Sex=male (95% CI)	133.18 (130.34 to 135.91)	133.92 (132.49 to 135.34)
WIMD (95% CI)*		
1	−114.34 (−118.59 to –109.94)	NA
2	−71.09 (−75.33 to –66.99)	NA
3	−39.73 (−44.15 to –35.34)	NA
4	−19.24 (−24.01 to –14.68)	NA
Maternal education (95% CI)†		
Lower secondary education, second stage of basic education	NA	0.54 (−7.08 to 8.48)
Upper secondary education	NA	86.16 (78.48 to 93.76)
Postsecondary non-tertiary education	NA	113.15 (71.9 to 155.69)
First stage of tertiary education, not advanced research qualification	NA	124.25 (116.21 to 131.96)
Second stage of tertiary education, advanced research qualification	NA	142.84 (124.75 to 159.22)
Not known	NA	−32.11 (−52.59 to –10.55)

*WIMD, where 1=most deprived and 5=least deprived, reference level WIMD=5.

†Reference level Maternal education= Primary education, first stage of basic education.

CF, cystic fibrosis; NA, not available; WIMD, Welsh Index of Multiple Deprivation.

#### Findings in the Danish population

In the Danish population, babies with a CF diagnosis were on average born about 3 days earlier than babies with no CF (−0.41 weeks; 95% CI −0.55 to −0.25 weeks). Parity and sex had negligible effects on gestational age, whereas gestational age increased by about 2 days when comparing babies born to mothers with primary education and those born to mothers with second stage tertiary education (table 2).

In total, babies with CF in the Danish population were 178.8 g (95% CI 134.47 to 225.43 g) lighter than babies with no CF. Approximately 35% of the effect of CF on birth weight was mediated by gestational age. First-born and female babies were significantly lighter than not first-born and male babies and birth weight increased by almost 150 g when comparing babies from mothers with primary education with those born to mothers with second stage tertiary education (table 3).

#### Findings in the Welsh population

In the Welsh population, CF-coded babies were born approximately 4 days earlier than non-CF-coded babies (−0.57 weeks; 95% CI −0.83 to −0.33 weeks). First-borns were found to be born slightly earlier than non-first-borns. Sex had a negligible effect on gestational age. The estimated increase in gestational age for babies from the least compared with the most deprived mothers was <1 day (see table 2 for details).

CF-coded babies in Wales were estimated to be born 210.08 g (95% CI 141.5 to 281.97 g) lighter than non-CF-coded babies with approximately 39% of the effect being due to reduced gestation. First-borns and females were significantly lighter than non-first-born and male babies. Birth weight increased by approximately 115 g when comparing the most with the least deprived quintile of babies (table 3).

### Robustness tests and additional analyses

Although no interaction effects between CF and any of the covariates were statistically significant (p<0.05), we found that the interaction between CF and first-born status was only marginally above the threshold level in both populations (see online supplementary material section S.5).

We found a significant interaction term between CF and gestational age in the birth weight submodel for the Danish population. However, the estimated direct and indirect effects of CF did not change markedly when this interaction term was introduced (see online supplementary material section S.6 for details). Results were similar in analyses including only one baby from each mother in both countries (online supplementary material section S.7). Repeating the analysis with additional covariates or using a stricter definition of CF cases did not markedly change our results (online supplementary material section S.7).

On the basis of simulations using our final model, we found that the probability for babies with CF to be born with low birth weight was between 1.3 and 1.8; and between 1.2 and 2.1 times that of babies with no CF in Wales and Denmark, respectively, and depended on sex, first-born status, deprivation and gestational age. The ratio between the probabilities of being born low birth weight for babies with  CF and babies with no CF increased with increasing gestational age. Being female, first-born or from a more deprived area increased the probability of being born with low birth weight only slightly and did not significantly affect the ratios between babies with CF and babies with no CF. See online supplementary material section S.8 for the effect of covariate values on the probabilities for a baby with CF to be born with low birth weight and the estimated ratios of the probabilities for babies with CF compared with babies with no CF.

## Discussion

In a whole population linkage study in Wales and Denmark, we showed that babies coded as having CF are born on average about 200 g lighter than babies with no CF. This is a large difference at a population level, whereby the total effect of CF on birth weight is similar to the impact of maternal smoking during pregnancy.^27^ Babies with CF are born on average about half a week earlier, but this only accounts for around 40% of the total effect of CF on birth weight, suggesting a significant direct biological impact of CF on intrauterine growth. Babies from socially disadvantaged backgrounds are significantly lighter than those from more affluent/more educated families, but this effect was the same in babies with CF and babies with no CF. Similarly, female babies are born significantly lighter than male babies and first-borns are born lighter than non-first-borns.

### Comparison with other studies

Our large study looking at births from two separate countries corroborates previous smaller studies that suggested a difference in birth weight between babies with CF and babies with no CF.^1 7–14^ Festini *et al* 7 compared perinatal data from 70 children with CF in Tuscany, Italy with regional population samples over an 11-year period. The authors found that overall babies with CF were born 246.2 g lighter (95% CI 129.8 to 362.5 g) than babies with no CF. In babies born at term (>37 weeks gestation), the difference was 205.7 g (95% CI 95.4 to 315.9 g). In another study, Darrah *et al* 1 compared the birth weight of 79 patients with CF born at term with pancreatic insufficiency and cared for at the Cystic Fibrosis Center at Rainbow Babies and Children’s Hospital in Cleveland, Ohio between 1975 and 2005 with the national average. In the study population, male babies with CF weighed an average 3239.80 g (SD=367.83 g, N=40) compared with a national average of 3530.20 g; female babies with CF weighed 3142.94 g (SD=422.75 g, N=39) compared with a national average of 3399.19 g. Recently, Ramos *et al* 13 conducted a study in Washington State comparing the birth weight of 170 babies with CF with that of 3400 babies with no CF matched by birth year and born between 1996 and 2013. They found mean birth weights of 3031 g (SD=759 g) and 3387 g (SD=581 g) for babies with and without CF, respectively. Earlier studies had found comparable results.^10–12^

Our results from Denmark and Wales show that the difference in birth weight between babies with CF and babies with no CF is about 200 g, similar to the estimates from these smaller studies. We extend these findings to assess the contribution of gestational age to explaining these effects. Our study highlights for the first time that there is a biological effect of CF on birth weight distinct from an impact of CF on gestational age. Different plausible biological explanations have been suggested, one of which being an association between cystic fibrosis transmembrane conductance regulator (CFTR) gene mutation and reduced levels of insulin-like growth factor 1 (IGF1). This association has been demonstrated in CF pig models, with reduced levels of IGF1 associated with reduced bone length and bone mineral content at birth.^28^ The same study also showed that new-born humans with CF also have reduced levels of IGF1. In addition, a study in mice with CF found strong correlations between IGF1 levels and weight.^29^ However, the study also showed that growth deficiency in mice with CF was evident late in gestation whereas, in contrast to the pig model, IGF1 levels were comparable to those in control mice prenatally and at birth but reduced at 3 weeks. It is therefore unlikely that reduced IGF1 levels are the sole cause for reduced weight at birth in CF. The study was carried out using mice with CF as well as gut-corrected mice with CF that did not present intestinal obstruction. Results were equivalent in both types of mice with CF with similar growth retardation compared with control, thus also ruling out a prominent role of intestinal obstruction in growth inhibition in mice with CF. Further investigation into a possible role of the placenta in CF-related growth deficiency showed that although aquaporin expression was altered in mice with CF, there was no evidence of an effect on placental fluid exchange.^29^ Further research is needed to fully understand the effect of CFTR mutations on placental function to assess its involvement in prenatal development in CF. Further studies are also needed to assess the impact of CF on different aspects of growth, for instance, birth length, which is not routinely collected in population registry data.

### Clinical implications

More research is needed to assess the prognostic value of birth weight for subsequent outcomes in CF. Nutritional status and growth in the early years are closely linked to lung function, which subsequently influences survival in CF.^2–5^ In a study of 79 patients with CF, birth weight has been shown to be associated with pulmonary function at age 6 and 10 years with FEV_1_% at age 6 increasing by an estimated 1% per additional 100 g birth weight.^1^

Within the CF study population, we found an average difference in birth weight of over 100 g between babies from the least and the most deprived families in both Wales and Denmark. Children with CF from the most deprived areas in the UK have been found to weigh less, be shorter, have a lower body mass index, be more likely to have chronic *Pseudomonas aeruginosa* infection and have lower lung function compared with children with CF from the least deprived areas.^15^ Similar results have also been found in the USA.^30^ Our study suggests that these differences may to some extent be explained by differences in birth weight. This indicates that social inequalities in CF outcomes may start in the intrauterine period.

### Strengths and limitations

A key strength is that our study made use of routinely collected data, which led to an unselected population-based cohort of around 2.2 million babies with 852 CF cases. A further strength is that we were able to carry out the analysis in two countries with remarkably consistent findings in both national populations. A wide range of information is collected in the Welsh and Danish registry linkages, allowing us to adjust for a range of variables in our analysis. A further strength compared with previous studies is that we have used modern methods for causal mediation analysis to better understand the role of gestational age in the pathway from CF to low birth weight. Misclassification of CF cases is a potential limitation of our analysis. CF is a lifelong condition that requires intensive healthcare support over the patient’s life, and all patients can be expected to receive hospital-based care at some point. It is therefore likely that all true CF cases are captured in both countries. In Wales, we were able to identify cases on the basis of both hospital inpatient and outpatient data and general practice records. In addition, a universal new-born screening programme was introduced in the UK (but not in Denmark) in 2007 leading to early diagnosis of CF in Wales and capture in the CARIS dataset. Any misclassification of CF cases identified in routine administrative datasets is expected to be independent of birth weight and gestational age and would, as such, lead to a conservative estimate of the effects on birth weight and gestational age. Robustness tests, selecting cases most likely to be true CF cases showed similar results to our main analysis.

In order to be able to estimate the direct and indirect effects of CF on birth weight, we made four assumptions to ensure identifiability of the natural indirect effect (see online supplementary material section S.6). If these assumptions do not hold there is the potential to introduce bias in the estimated effects, which is a well-known problem in causal inference.^31 32^ In order to minimise the risk of biased estimates, we included many of the well-established factors that affect birth weight and gestational age^33^ in the analysis and conducted robustness tests in which we included further variables that were only available in subsets of the study population. Our results did not change, increasing our confidence that omission of these variables in the main analysis did not affect our findings markedly. However, we were not able to adjust for variables such as prepregnancy body mass index, and weight gain during pregnancy, which are not collected in the registry data that could potentially lead to mediator-outcome confounding. Similarly, we were not able to adjust for ethnicity, which may be a potential confounder and may plausibly modify the relationship between CF and birth weight. The non-white populations of both Wales and Denmark are small (7% and 10%, respectively) and they may therefore be ill-suited to give insights into CF-ethnicity interactions. Since non-white ethnicity is associated with worse outcomes in CF,^34–36^ it may, however, be of interest to explore this in future studies in other populations. One further point to consider is that gestational age is prone to measurement error or interval censoring. This may have the effect of reducing the association between gestational age and birth weight and therefore the indirect effect of CF while increasing the direct effect.^37^ Due to the size of our study population, we do not believe that this is a major concern in this study; it should, however, be taken into account when interpreting the findings.

### Implications for research and practice

Birth weight and gestational age are not currently collected in most CF registry datasets, including the UK, the US and Danish databases. Based on our study results, we recommend the addition of birth weight to the list of variables collected in CF registries. This will allow further longitudinal studies to understand the association of birth weight with trajectories of nutritional status and lung function and its association with survival. Further research is also needed to understand the biological mechanisms that lead to reduced intrauterine growth in babies with CF compared with babies with no CF. Finally, our study has shown the merits of data linkage, without which we would not have been able to adjust for all the important confounders and identify the direct and indirect effects. In order to further our understanding of disease progression in CF, linkage of CF registries to other routinely collected data sources should be considered.

In conclusion, our findings from the analysis of national populations in Wales and Denmark suggests that CF has a significant effect on birth weight, and that this is only partially due to an effect on gestational age.
