# Supplementary material for: Impact of cystic fibrosis on birthweight: a population based study of children in Denmark and Wales
Source: Thorax. 2018 Jul 19;74(5):447–54. doi: 10.1136/thoraxjnl-2018-211706 (PMC6484694; doi:10.1136/thoraxjnl-2018-211706)
Supplement: Supplementary data [file thoraxjnl-2018-211706supp001.pdf]

Impact of cystic fibrosis on birthweight: a population based study of children in Denmark and Wales

Supplementary Material

S.1 Data set generation and cleaning

Denmark

Since 1968 all children born in Denmark have been issued with a unique personal identification number, the Central Person Registry (CPR) number. Using this number it is possible to link a number of demographic, socio-economic and birth datasets, and vital statistics for each individual across several nationally collected registers, and to use these data for research.

All individuals born between 1980 and 2010 were identified from the Danish Medical Birth Register. Data were extracted from the Danish Medical Birth Register for date of birth, birth weight, gestational age at birth, multiplicity, sex, parity, mode of delivery, maternal age at birth and maternal smoking during pregnancy (available from 1996 onwards). Data on pre-eclampsia (ICD 8 code 637, ICD 10 code O14 and O15) in index pregnancy and diabetes during index pregnancy (ICD 8 code 250, ICD 10 code O24) were extracted from the National Patient Register. Maternal highest attained education in year before index conception was extracted from the Education Register.

Babies marked as Singletons but where the mother's age at birth, parity and the gestational age of the babies were identical between two or more individuals were removed from the analysis under the assumption that these babies were not actually singletons.

Parity was defined as the number of pregnancies that lasted beyond 22 weeks including the index pregnancy. Therefore babies with parity of zero and 99 were removed from the data set. We found that parity alone could not be used to uniquely identify first-borns, therefore in those cases where more than one baby was indicated as first born to the same mother, the mother's age at birth was used to establish the true first born.

Wales

Every person in Wales who is registered with the National Health Service (NHS), is allocated a NHS number. This number is encrypted to create an Anonymized Linkage Field (ALF) used to link across administrative and healthcare datasets. We accessed ALFs for all children born in Wales between 1998 and 2015 and their mothers through the National Community of Child Health (NCCH) Database. We used the ALFs for the child and mother to extract individual-level anonymised data from five additional data sources including the Congenital Anomaly Register and Information Service (CARIS), Patient Episode Database for Wales (PEDW), Welsh Longitudinal General Practice data (WLGP), the Annual District Birth Extracts (ADBE; also known as the Office of National Statistics (ONS) birth register), and the Welsh Demographics Service Dataset (WDSD).

All individuals were identified in the National Community of Child Health / National Child Cohort for Health (NCCH) and their Anonymised Linkage Field (ALF) were extracted together with gestational age at birth, sex, twin status, ALF of the mother and previous live and still births. Linkage with the following databases allowed the extraction of all data used in the analysis:

- **CARIS** (Congenital Anomaly Register and Information Service; CF diagnosis at birth (ICD 10 code E84), multiplicity);
- **WLGP** (Welsh Longitudinal General Practice data; CF diagnosis after birth (READ codes 1264. , 66k, 66k0. , 9No7., C370), mother's smoking status, mother's week of birth);
- **PEDW** (Patient Episode Database for Wales; CF diagnosis after birth (ICD 10 codes E84), Meconium ileus (ICD 10 code =E841, E84.11, E84.19, P76, , K56.6);
- **ADBE**(Annual District Birth Extracts from Office of National Statistics (ONS); birth weight, week of birth, sex);
- **WDSD** (Welsh Demographics Service Dataset; WIMD scores);
- **NCCH (**National Community of Child Health/ National Child Cohort for Health; gestational age, parity**)**

The mother's age at birth was calculated as difference in days between week of birth of the baby and week of birth of mother divided by 365.25. Whether a baby was first born was established from the number of previous live births the mother had had (zero previous live births indicated that the baby was first born).

Both populations

Babies from multiple births were excluded from the analysis.

Babies with birth weights less than and including 100g or more than and including 7kg were excluded as these were considered unrealistic and therefore assumed to be data entry errors. Similarly, those babies with gestational age less than and including 21 weeks or more than and including 45 weeks were removed from the data set.

S.2 Mixture Sub-populations

Figures S1 and S2 show the distribution of gestational age and birthweight in Wales and Denmark, respectively, as well as the fitted model. One sub-population accounted for 91.01% (95% CI: 90.88%, 91.1%) and 88.9% (95% CI: 88.78%, 89.19%) of births in Denmark and Wales, respectively, and will henceforth be referred to as the primary component or sub-population. In Denmark, the primary component had a higher birthweight and gestational than the secondary components (3563.03g and 40.01weeks compared to 2708.69g and 36.44weeks). Similarly, in Wales, the primary sub-population had an average birthweight of 3460.18g and an average gestational age of 39.71 weeks. The secondary component had a lower birthweight and gestational age, estimated to be 2758.82g and 36.44 weeks, respectively. In both countries, the variances around the mean birthweight and gestational age were larger in the secondary than in the primary sub-population. In Denmark, the standard deviations of birthweight and gestational age in the primary component were 492.53g and 1.55weeks, respectively, compared to 751.13g and 10.92weeks in the secondary population. In Wales, the standard deviations

in the primary population were 472.55g and 1.24 weeks whereas in the secondary population they were 818.04g and 3.32 weeks.

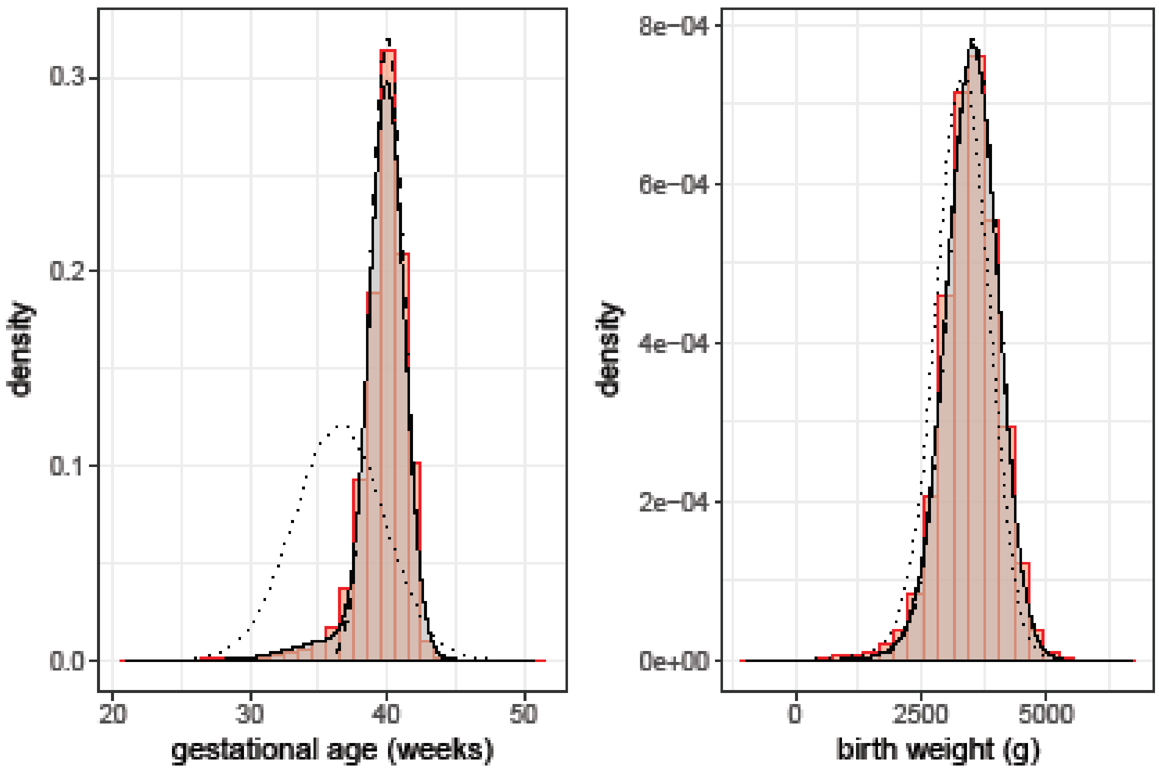

Figure S1: Distribution of gestation age (left panel) and birth weight (right panel) in the Danish study population is shown by the red histogram. The dashed line indicates the fitted distribution of the primary sub-population, the dotted line the distribution of the secondary sub-population and the solid line and the grey shaded region the fitted distribution for the whole population. The raw data and the fitted models represent the entire population and do not distinguish between CF and non-CF cases.

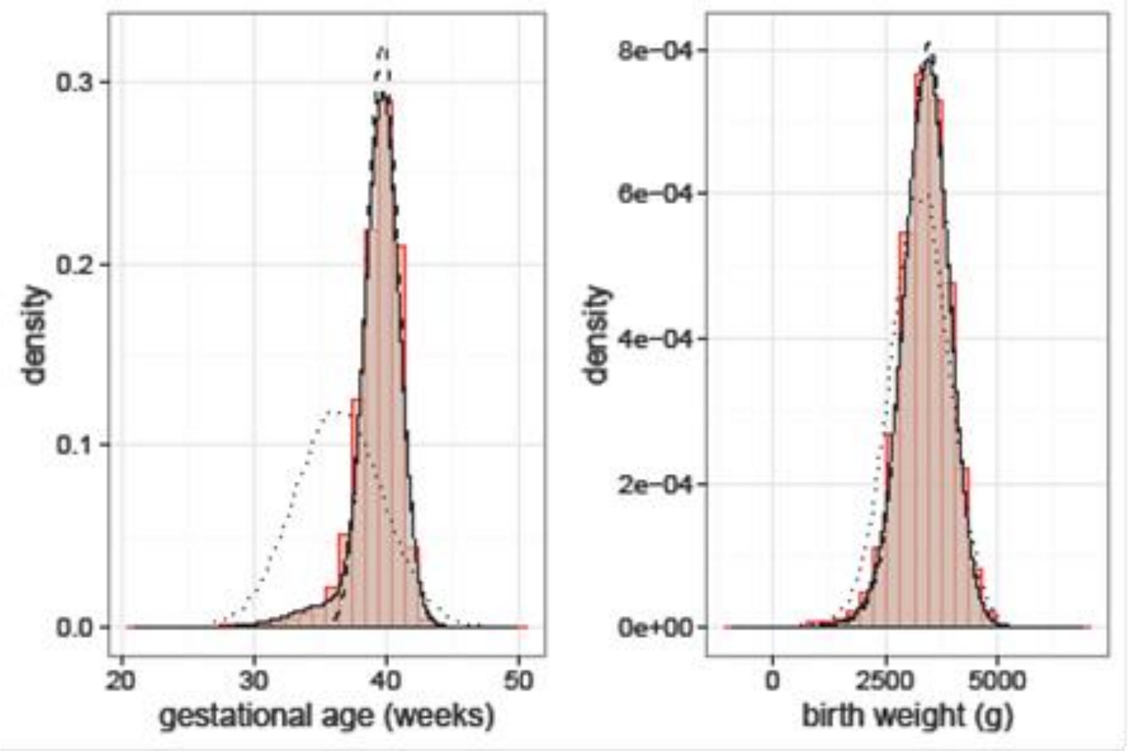

Figure S2: Distribution of gestation age (left panel) and birth weight (right panel) in the Welsh study population is shown by the red histogram. The dashed line indicates the fitted distribution of the primary sub-population, the dotted line the distribution of the secondary sub-population and the solid line and the grey shaded region the fitted distribution for the whole population. The raw data and the fitted models represent the entire population and do not distinguish between CF and non-CF cases.

S.3 Testing main effects for significance by backward elimination

The full model was initially fitted and main effects terms were removed individually from the sub-model for birthweight, the sub-model for gestational age and both sub-models. Likelihood ratio tests were carried out to assess the significance of the covariates. All covariates were found to be significant at the 5% level. Tables S1 and S2 show the deviances and p-values from the likelihood ratio tests for the Welsh and the Danish populations, respectively.

Table S1: P-values from the Likelihood ratios tests of significance of the individual covariates included in the model of birthweight and gestational age in the Danish population.

Covariate	Removal from gestational age sub-model		Removal from birthweight sub-model		Complete removal	
	deviance	p-value	deviance	p-value	deviance	p-value
Gestational age	NA	NA	296373.04	~0	NA	NA
CF	41.46	9.93E-10	44.64	2.03E-10	75.6	1.49E-15
sex	262.25	1.13E-57	37542	~0	38356.13	~0

First born status	4132.04	~0	22819.81	~0	26776.53	~0
Maternal education	4164.93	~0	19514.45	~0	22819.05	~0

Table S2: Deviances and p-values from the Likelihood ratios tests of significance of the individual covariates included in the model of birthweight and gestational age in the Welsh population.

Covariate	Removal from gestational age sub-model		Removal from birthweight sub-model		Complete removal	
	deviance	p-value	deviance	p-value	deviance	p-value
Gestational age	NA	NA	66553.89	~0	NA	NA
CF	28.02	8.24e-07	21.79	1.85e-05	50.08	3.48e-10
sex	126.55	3.31e-28	9936.81	~0	10183.42	~0
First born status	3652.59	~0	7223.36	~0	11004.04	~0
WIMD	289.68	6.47e-58	3721.01	~0	3928.51	~0

S.4 Estimated effects in the primary and secondary populations

Findings in the Danish Population

Primary Sub-population

In the primary sub-population, babies with CF were estimated to be born about a quarter of a week earlier than non-CF babies (-0.26 weeks 95%CI -0.39 to -0.13). The difference in gestational age between first-borns and non-first-borns was estimated to be 0.12 weeks (95%CI 0.12 to 0.13). While the effect of sex was negligible, differences in maternal education were estimated to have effects of up to a quarter of a week increase in gestational age with increased education (see Table S3 for details).

The birthweight of babies with CF in the primary component was estimated to be 107.65g (95%CI 70.16g to 147.03g) less than that of non-CF babies of the same gestational age, sex, first born status and maternal education. Per week gestation, birth weight was estimated to increase by 137.72g (95%CI 137.03g to 138.64g). This leads to an additional indirect effect of CF on birth weight through gestational age of -36.34g (95%CI -54.2g to -18.2g). In total, CF babies in the primary sub-population were therefore estimated to be 143.99g lighter (95%CI 100.28g to 190.09g) than non-CF babies. Females and first-borns were estimated to be lighter than males and not-first-borns and birthweight was estimated to increase with increasing maternal education (see Table S4 for details).

Secondary Sub-population

In the secondary sub-population, CF babies were estimated to be born between one and two and a half weeks earlier than non-CF-babies (-1.84 95%CI -2.52 to -1.03). Sex and first-born status were also found to have large effect sizes with males and first-borns being born earlier. Maternal education did appear to affect gestational age, in particular babies born to mothers with education level 5 or 6 were

born later than those born to mothers with education level 1, however, there was no clear increasing trend of gestational age with maternal education (see Table S3).

The direct effect of CF on birthweight was estimated to be -201.48g (95%CI -298.02g to -100.15g). Birthweight was estimated to increase by 178.85g (95%CI 177.05g to 180.17g) per week gestation leading to an additional indirect effect of CF on birthweight of -329.5 (95%CI: -449.34g, -184.2g). Thus, in total, CF babies in the secondary sub-population were estimated to be 530.98g (95%CI:353.27g to 688.07g) lighter than non-CF babies. First-borns and females were again estimated to be lighter than non-first-borns and males. The effect of maternal education on birthweight in the secondary subpopulation was unclear (see Table S4 for details).

Findings in the Welsh population

Primary Sub-population

Babies with CF in the primary sub-population were found to be born about half a week earlier (-0.46 weeks 95% CI -0.66 to -0.27) than non-CF babies. The other covariate with big effect size was first-born status with first-borns born a quarter week later (0.26 weeks 95% CI 0.25 to 0.26) than not first-borns. The effects of sex and deprivation scores on gestational age were negligible.

The birthweight of CF babies in the primary component was estimated to be 140.68g (95% CI 79.08g to 202.88g) lower than that of non-CF babies of the same gestational age, sex, first born status and deprivation quintile. Per week gestation, birth weight was estimated to increase by 135.96g (95%CI 134.25g to 137.51g). This leads to an additional indirect effect of CF on birthweight through gestational age of -62.25g (95% CI -89.75g to -36.81g). Thus, in total, CF babies in the primary population were born 202.92g (95%CI 136.29g to 269.63g) lighter than non-CF babies. In addition female babies and first-borns were estimated to be lighter than males and babies that are not first-borns. Birth weight was estimated to decrease with increasing deprivation with the gap between the most and the least deprived being -117.22g (95%CI: -121.91g, -112.47g).

Secondary Sub-population

In the secondary sub-population the effect of CF on gestational age was estimated to be -1.55 weeks (95%CI -3.3 to 0.16)). Sex, first-born status and socio-economic status were also found to have large effect sizes with males, first-borns and more deprived children being born earlier.

The direct effect of CF on birthweight was estimated to be -19.04g (95%CI -240.82g to 181.27g). Birth weight was estimated to increase by 168.75g (95%CI: 164.17g, 173.17g) per week gestation leading to an indirect effect of -248.65g (95%CI -555.78g to 27.13g). In total, CF babies in the secondary

population were thus estimate to be 267.69g (96%CI -664.77g to 67.32g) lighter than non-CF babies. The effects of gender, first-born status and deprivation were estimated to be smaller in this sub-population compared to the primary one, yet showing the same trends (see Table S4).

Table S3: Parameter estimates (95% confidence intervals) for the covariates in gestational age sub-model.

	Wales		Denmark	
	Primary component	Secondary component	Primary component	Secondary Component
CF	-0.46 (-0.66, -0.27)	-1.5 (-3.3, 0.16)	-0.26 (-0.39, -0.13)	-1.84 (-2.52, -1.03)
First born	0.26 (0.25, 0.26)	-0.58 (-0.65, -0.5)	0.12 (0.12, 0.13)	-0.89 (-0.95, -0.86)
Sex =male	-0.036 (-0.028, -0.044)	-0.26 (-0.18, -0.33)	-0.02 (-0.02, -0.02)	-0.28 (-0.31, -0.23)
WIMD [1]				
1	-0.058 (-0.071 , -0.045)	-0.52 (-0.64 , -0.41)	NA	NA
2	-0.014 (-0.027 , 8.7e-05)	-0.34 (-0.45 , -0.22)	NA	NA
3	0.0039 (-0.0097 , 0.018)	-0.21 (-0.33 , -0.094)	NA	NA
4	0.018 (0.0049 , 0.032)	-0.039 (-0.16 , 0.089)	NA	NA
Maternal education				
Lower secondary education, second stage of basic education	NA	NA	0.06 (0.03 , 0.09)	-0.42 (-0.7 , -0.11)
Upper secondary education	NA	NA	0.15 (0.13 , 0.18)	0.14 (-0.14 , 0.46)
Post secondary non tertiary education	NA	NA	0.24 (0.1 , 0.39)	0.71 (-0.99 , 2.11)
First stage of tertiary educ., not adv. research qualification	NA	NA	0.21 (0.19 , 0.24)	0.41 (0.12 , 0.7)
Second stage of tertiary educ., adv. research qualification	NA	NA	0.22 (0.16 , 0.27)	0.97 (0.39 , 1.4)
Not known	NA	NA	0.23 (0.16 , 0.3)	0.24 (-0.48 , 0.97)
[1] Welsh Index of Multiple Deprivation where 1=most deprived and 5=least deprived				

Table S4: Parameter estimates (95% confidence intervals) for the covariates in birthweight sub-model.

	Wales		Denmark	
	Primary component	Secondary component	Primary component	Secondary Component
Gestational age (g per week)	135.96 (134.25 , 137.51)	168.75 (164.17 , 173.17)	137.72 (137.03 , 138.64)	178.85 (177.05 , 180.17)
CF direct	-140.68 (-202.88 , -79.08)	-19.04 (-240.82 , 181.27)	-107.65 (-147.03 , -70.16)	-201.48 (-298.02 , -100.15)

CF indirect	-62.25 (-89.75 , -36.81)	-248.65 (-555.78 , 27.13)	-36.34 (-54.2 , -18.2)	-329.5 (-449.34 , -184.2)
CF total	-202.92 (-269.63 , -136.29)	-267.69 (-664.77 , 67.32)	-143.99 (-190.09 , -100.28)	-530.98 (-688.07 , -353.27)
First born	-119.27 (-122.32 , -116.22)	-82.48 (-96.64 , -67.69)	-120.34 (-122.06 , -118.91)	-45.16 (-53.63 , -40.14)
Sex =male	141.24 (144.12, 138.15)	68.3 (82.61, 55.17)	140.15 (138.57, 141.65)	70.91 (64.3, 77.53)
WIMD [1]				
1	-117.22 (-121.91 , -112.47)	-91.09 (-114.29 , -67.74)	NA	NA
2	-71.79 (-76.61 , -67.02)	-65.41 (-88.72 , -44.07)	NA	NA
3	-40.07 (-44.9 , -35.34)	-36.95 (-59.65 , -13.71)	NA	NA
4	-17.63 (-22.6 , -12.68)	-32.21 (-56.85 , -9.3)	NA	NA
Maternal education				
Lower secondary education, second stage of basic education	NA	NA	9.92 (0.68 , 18.93)	-94.32 (-138.58 , -47.79)
Upper secondary education	NA	NA	97.86 (89.08 , 106.75)	-32.25 (-77.78 , 12.14)
Post secondary non tertiary education	NA	NA	135.88 (90.93 , 181.45)	-116.8 (-309.93 , 151.64)
First stage of tertiary educ., not adv. research qualification	NA	NA	136.79 (127.5 , 145.7)	-2.64 (-50.51 , 40.4)
Second stage of tertiary educ., adv. research qualification	NA	NA	163.17 (144.03 , 180.77)	-62.87 (-143.86 , 9.83)
Not known	NA	NA	-34.96 (-57.16 , -10.48)	-3.22 (-131.57 , 110.38)
[1] Welsh Index of Multiple Deprivation where 1=most deprived and 5=least deprived				

S.5 Testing significance of interaction terms

Interaction effects between CF and any of the other main effects were tested for significance using the likelihood ratio test. None of the interaction effects were found to be significant at the 5% level. Tables S5 and S6 show the resulting deviances and p-values.

In both populations, the interaction effect between CF and first-born status on gestational age was only marginally not significant and may therefore be of interest. We thus fitted the model in both populations including this interaction term and found that it did not significantly change the estimated effects CF has on birthweight. As we could also not find a possible biological explanation for this phenomena we have not included this in the main article.

Table S5: Deviances and p-values from the likelihood ratio tests assessing the significance of interaction effects of CF with any of the other main effects in the model for the Danish population.

Interaction term	Adding interaction term to gestational age sub-model		Adding interaction term to birth weight sub-model	
	deviance	p-value	deviance	p-value
CF:sex	~0	~1	0.42	0.81

CF:first born	5.78	0.06	~0	~1
CF:education	24.1	0.93	4.81	0.96
CF:gestational age	NA	NA	8.25	0.02

Table S6: Deviances and p-values from the likelihood ratio tests assessing the significance of interaction effects of CF with any of the other main effects in the model for the Welsh population.

Interaction term	Adding interaction term to gestational age sub-model		Adding interaction term to birth weight sub-model	
	deviance	p-value	deviance	p-value
CF:sex	0.75	0.69	0.16	0.92
CF:first born status	6.03	0.05	1.12	0.57
CF:WIMD	1.75	0.99	1.33	1

S.6 Interaction between CF and gestational age in the birthweight sub-model

In the Danish population we found a significant interaction effect between CF and gestational age on birthweight (see Table S7), meaning that the effect of gestational age on birthweight differs between CF and non-CF babies. Taking into account this interaction term, the effects of CF and all covariates on gestational age remained unchanged, as did the effects of first-born status, sex, and maternal education on birthweight (see Table S9). Table S8 summaries the effects of gestational age and the direct and indirect effects of CF on birthweight when taking into account the interaction term (see below for details on the derivation of the direct and indirect effects). The direct and indirect effects of CF are comparable to the results when no interaction effect between CF and gestational age is taken into account.

Table S7: Deviance and p-values from likelihood ratio test used to assess possibility of an interaction effect of CF and gestational age on birthweight

country	deviance	p-value
Denmark	8.25	0.02
Wales	0.05	0.97

Calculation of direct and indirect effects from the regression parameters when there is an exposure-mediator interaction

The information given in this section is a restatement of previous findings. For more details and the derivation of the estimators, see [1, 2].

The estimation of direct and indirect affects requires four key assumptions:

1) No unmeasured exposure-outcome confounding
2) No unmeasured mediator-outcome confounding
3) No unmeasured exposure-mediator confounding
4) No variable exists, that is affected by the exposure and confounds the mediator-outcome relationship

Under these assumptions, the natural direct and indirect effect are identified and can be estimated as follows. Let *Y* denote the outcome of interest, *A* the exposure, *M* the potential mediator and *C* the

baseline covariates. A linear regression of the outcome Y on the exposure, mediator and covariates including an exposure-mediator interaction is then given by

$$E[Y|a,m,c] = \alpha_0 + \alpha_1 a + \alpha_2 m + \alpha_3 am + \alpha_4' c.$$

A linear regression of the mediator on the exposure and the baseline covariates is

$$E[M|a,c] = \beta_0 + \beta_1 a + \beta_2' c.$$

The natural direct and indirect effect are then given by

$$DE = (\alpha_1 + \alpha_3(\beta_0 + \beta_1 a^* + \beta_2' c))(a - a^*)$$

and

$$IE = (\alpha_2\beta_1 + \alpha_3\beta_1 a)(a - a^*)$$

where a and a^* are two different levels of exposure; in our example a =1 and a^* =0 indicate CF and no CF, respectively. Note that when there is no exposure-mediator interaction (i.e. when $\alpha_3 = 0$) then the direct effect is estimated by α_1 and the indirect effect is estimated by $\alpha_2\beta_1$.

The estimated direct effect is dependent on baseline covariates. Table 8 below gives the direct effect at reference covariate levels, for females, and individuals with education level 6 (compared to males and education level 1, which are the reference levels). The estimates only vary negligibly. Table 9 gives the estimates for the remaining parameters.

Table S8: Estimated effects (95% confidence intervals) of gestational age and CF on birth weight when accounting for CF-gestational age interaction in the Danish population.

	Primary Component	Secondary component	Total population
Gestational age (g per week)	137.73 (137.06 , 138.6)	178.85 (177.3 , 180.15)	141.43 (140.87 , 142.2)
Gestational age – CF interaction (g per week in CF babies)	38.5 (7.08 , 71.74)	18.67 (-14.32 , 73.74)	36.72 (-139.75 , -61.67)
Total effect of gestational age in CF babies (g per week)	176.23 (145.05 , 209.6)	197.52 (164.74 , 252.41)	178.14 (290.93 , 351.72)
CF indirect	-47.5 (-73.6 , -22.92)	-369.17 (-511.17 , -227.74)	-76.41 (-104.39 , -48.41)
natural direct effect at covariate reference levels	-109.8 (-150.92 , -71.96)	-122.79 (-289.56 , 108.55)	-110.97 (-148.26 , -73.58)
natural direct effect for males	-110.56 (-151.29 , -72.58)	-127.93 (-288.69 , 91.35)	-112.12 (-149.25 , -75.19)
natural direct effect for education level 6	-101.48 (-144.11 , -62.29)	-104.71 (-300.15 , 175.74)	-101.77 (-139.75 , -61.67)

Table S9: Table of parameter estimates (95% confidence intervals) for the birthweight sub-model in the Danish population when the interaction between CF and gestational age is taken into account

	Primary Component	Secondary component
First born	-120.32 (-122.04 , -118.83)	-45.23 (-53.72 , -40.28)
Sex =male	140.15 (138.7 , 141.65)	70.86 (64.84 , 77.88)
Maternal education		

	Lower secondary education, second stage of basic education	9.95 (1.4 , 18.71)	-94.64 (-139.32 , -47.95)
	Upper secondary education	97.89 (89.46 , 106.27)	-32.59 (-77.48 , 14.69)
	Post secondary non tertiary education	136.07 (86.99 , 180.37)	-117.84 (-299.27 , 163.96)
	First stage of tertiary educ., not adv. research qualification	136.82 (128.22 , 145.16)	-3.01 (-49.1 , 41.65)
	Second stage of tertiary educ., adv. research qualification	163.21 (144.6 , 181.25)	-63.38 (-141.21 , 12.06)
	Not known	-34.95 (-59.08 , -10.88)	-3.69 (-128.41 , 121.98)

S.7 Results from further Robustness tests

One child per mother

The analyses including only one baby from each mother resulted in a slightly higher point estimate of the direct effect of CF on birthweight in Wales and slighter lower point estimate in Denmark, though the estimates were within the previously stated confidence limits. All other effects also remained consistent with the results presented previously, with the exception for first-born status the effect of which decreased in both populations. Tables S10 and S11 show the parameter estimates and associated standard errors found in the analysis. For comparison, the tables also contain the estimates and standard errors from the analysis shown in the main paper.

Table S10: Parameter estimates (standard errors) for the model fit to the data of one child per mother in the Danish population. Values are rounded to two digits.

	Sensitivity analysis		Control analysis	
Covariate	primary component	secondary component	primary component	secondary component
Gestational age				
CF	-0.34 (0.08)	-1.95 (0.49)	-0.26 (0.06)	-1.84 (0.42)
Sex (=male)	-0.02 (0)	-0.3 (0.03)	-0.02 (0)	-0.28 (0.02)
first born	0.13 (0)	-0.64 (0.03)	0.12 (0)	-0.89 (0.02)
Maternal education				
Lower secondary education, second stage of basic education	0.1 (0.02)	-0.42 (0.18)	0.06 (0.01)	-0.42 (0.13)
Upper secondary education	0.17 (0.02)	-0.05 (0.18)	0.15 (0.01)	0.14 (0.13)
Post secondary non tertiary education	0.28 (0.09)	0.21 (0.97)	0.24 (0.07)	0.71 (0.72)
First stage of tertiary educ., not adv. research qualification	0.24 (0.02)	0.15 (0.18)	0.21 (0.01)	0.41 (0.13)
Second stage of tertiary educ., adv. research qualification	0.21 (0.05)	0.64 (0.44)	0.22 (0.03)	0.97 (0.29)

Not known	0.27 (0.05)	0.08 (0.44)	0.23 (0.03)	0.24 (0.33)
Birthweight				
CF	-89.18 (17.65)	-268.09 (73.33)	-107.65 (13.75)	-201.48 (29)
Sex (=male)	132.64 (18.74)	68.79 (36.98)	140.15 (20.54)	70.91 (55.48)
first born	-25.52 (25.88)	-7.86 (62.03)	-120.34 (0.75)	-45.16 (3.15)
Maternal education				
Lower secondary education, second stage of basic education	8.12 (1.02)	-82.52 (3.8)	9.92 (0.82)	-94.32 (3.27)
Upper secondary education	73.62 (6.67)	-23.11 (28.34)	97.86 (4.52)	-32.25 (21.05)
Post secondary non tertiary education	123.77 (6.65)	-182.82 (28.29)	135.88 (4.5)	-116.8 (21.01)
First stage of tertiary educ., not adv. research qualification	97.99 (32.99)	-10.56 (129.04)	136.79 (24.9)	-2.64 (114.17)
Second stage of tertiary educ., adv. research qualification	80.17 (6.68)	-138.38 (28.4)	163.17 (4.52)	-62.87 (21.09)
Not known	-29.81 (16.59)	3.26 (66.68)	-34.96 (10.29)	-3.22 (46.56)
Gestational age	133.56 (1.04)	181.37 (3.98)	137.72 (11.83)	178.85 (55.03)

Table S11: Parameter estimates (standard errors) for the model fit to the data of one child per mother in the Welsh population. Values are rounded to two digits.

	Sensitivity analysis		Control analysis	
	Primary Component	Secondary Component	Primary Component	Secondary component
Gestational age				
CF	-0.31(0.14)	-0.79 (1.43)	-0.46 (0.09)	-1.47 (0.76)
Sex (=male)	-0.04 (0.01)	-0.25 (0.06)	-0.04 (0)	-0.26 (0.04)
First born	-1.25 (0.04)	-2.67 (0.39)	0.26 (0)	-0.58 (0.04)
WIMD				
1	-0.06 (0.01)	-0.59 (0.1)	-0.06 (0.01)	-0.52 (0.06)
2	0 (0.01)	-0.39 (0.11)	-0.01 (0.01)	-0.34 (0.06)
3	0.01 (0.01)	-0.19 (0.11)	0 (0.01)	-0.21 (0.06)
4	0.03 (0.01)	-0.03 (0.11)	0.02 (0.01)	-0.04 (0.07)
Birth weight				
CF	-198.01 (47.8)	45.26 (268.22)	-140.68 (30.83)	-19.04 (130.97)
Sex (=male)	147.8 (2.32)	75.25 (13.31)	141.24 (1.48)	68.3 (7.15)
First born	-51.46 (15.24)	-110.77 (69.2)	-119.27 (1.5)	-82.48 (7.24)
WIMD				
1	-135.56 (3.65)	-75.19 (21.24)	-117.22 (2.37)	-91.09 (11.61)
2	-83.3 (3.75)	-65.31 (22.05)	-71.79 (2.42)	-65.41 (11.86)
3	-48.65 (3.84)	-20.08 (22.68)	-40.07 (2.48)	-36.95 (12.2)
4	-22.42 (3.93)	-6.03 (23.63)	-17.63 (2.55)	-32.21 (12.7)
Gestational age	137.65 (1.03)	172.63 (2.63)	135.96 (0.66)	168.75 (1.48)

Additional covariates

Data on maternal smoking during pregnancy and age at birth were available on a subset of 119,712 individuals including 65 CF babies in Wales. In Denmark we had data on mode of delivery, diabetes

during index pregnancy, and pre-eclampsia during index pregnancy in addition to maternal age at birth and smoking during pregnancy in 1,032,281 individuals including 257 CF babies. Tables S12 and S13 show the parameter estimates and associated standard errors from fitting the model to the sub-population for whom the additional data were available. For comparison, the model was initially fitted to the same population without the additional covariates and then refitted adding in these covariates.

In Wales the estimates for the effects of CF on both gestational age and birthweight were lower in this sub-population than in the previously stated results but within the previously stated confidence limits. There was no significant difference between the estimates for the effect of CF from the model with and without the adjustment for smoking and maternal age. The only variable affected was deprivation, the effect of which on birthweight decreased substantially after maternal smoking during pregnancy and maternal age at birth were adjusted for. This is unsurprising as there are strong correlations between all three variables.

In Denmark the estimates for the effects of CF on gestational age and birthweight remained consistent with previous results after adjustment for the additional factors. Similar to the results from the Welsh analysis, maternal education was the only covariate affected by the inclusion of the additional factors.

Table S12: Parameter estimates (standard errors) for the model fitted to the sub population in Denmark for whom data was available on maternal smoking during pregnancy, maternal age at birth, diabetes during index pregnancy, pre-eclampsia during index pregnancy and mode of delivery (reference level: vaginal). Values are rounded to two digits.

	Analysis with adjustment for additional covariates		Analysis without adjustment for additional covariates	
covariate	primary component	secondary component	Primary component	Secondary component
Gestational age				
CF	-0.2 (0.09)	-1.02 (1.09)	-0.23 (0.09)	-1.15 (0.69)
Sex (=male)	-0.01 (0)	-0.26 (0.12)	-0.02 (0)	-0.21 (0.03)
first born	0.11 (0)	-0.83 (0.48)	0.12 (0)	-1.09 (0.03)
Maternal education				
Lower secondary education, second stage of basic education	0.12 (0.01)	-0.16 (0.02)	0.05 (0.01)	-0.41 (0.15)
Upper secondary education	0.18 (0.01)	0.17 (0.1)	0.14 (0.01)	0.04 (0.15)
Post secondary non tertiary education	0.23 (0.07)	0.34 (0.1)	0.2 (0.07)	0.29 (0.76)
First stage of tertiary educ., not adv. research qualification	0.23 (0.01)	0.3 (0.51)	0.2 (0.01)	0.12 (0.15)

Second stage of tertiary educ., adv. research qualification	0.23 (0.03)	0.69 (0.1)	0.19 (0.03)	0.58 (0.32)
Not known	0.19 (0.03)	-0.11 (0.22)	0.17 (0.04)	-0.08 (0.35)
smoking	-0.1 (0)	-0.56 (0.03)	NA	NA
maternal age	0.01 (0)	-0.02 (0.24)	NA	NA
Mode of delivery				
elective c-section	-1.39 (0.01)	-1.53 (0)	NA	NA
emergency c-section	0.17 (0.01)	-2.99 (0.04)	NA	NA
diabetes	-0.21 (0.28)	0.44 (0.02)	NA	NA
preeclampsia	-0.29 (0.25)	-3.36 (1.83)	NA	NA
Birthweight				
CF	-110.32 (103.57)	-95.18 (162.02)	-108.47 (12.77)	-95.22 (75.29)
Sex (=male)	139.78 (20.2)	73.24 (34.87)	137.52 (17.55)	75.08 (55.41)
first born	-159.9 (31.5)	-97.85 (77.19)	-149.52 (29.6)	-126.05 (117.81)
Maternal education				
Lower secondary education, second stage of basic education	91.62 (0.98)	18.79 (3.43)	42.87 (0.94)	-100.47 (5.39)
Upper secondary education	133.96 (5.68)	58.4 (21.52)	123.63 (5.37)	-57.72 (32.09)
Post secondary non tertiary education	140.71 (5.62)	-137.78 (21.35)	146.2 (5.32)	-342.16 (31.88)
First stage of tertiary educ., not adv. research qualification	143.24 (27.36)	69.35 (88.58)	155.35 (26.21)	-53.39 (138.41)
Second stage of tertiary educ., adv. research qualification	140.98 (5.65)	-22.7 (21.45)	168.25 (5.34)	-246.12 (31.98)
Not known	-67.38 (11.61)	59.28 (41.9)	-40.53 (10.98)	-70.51 (63.57)
gestational age	136.5 (1.08)	171.3 (3.63)	144.9 (0.98)	209.51 (5.51)
maternal age	0.02 (13.46)	-1.67 (52.4)	NA	NA
Mode of delivery				
elective cs	68.23 (0.12)	-366.44 (0.39)	NA	NA
emergency cs	86.1 (2.14)	-305.41 (7.27)	NA	NA
smoking	-169.26 (1.95)	-149.27 (4.38)	NA	NA
diabetes	-150.04 (1.21)	774.37 (4.12)	NA	NA
Pre-eclampsia	-1.94 (124.31)	-402.66 (323.99)	NA	NA

Table S13: Parameter estimates (standard errors) for the model fitted to the sub population in Wales for whom data was available on maternal smoking during pregnancy and maternal age at birth. Values are rounded to two digits.

	Analysis with adjustment for smoking and maternal age		Analysis without adjustment for smoking and maternal age	
Covariate	Primary Component	Secondary Component	Primary Component	Secondary Component
Gestational age				
CF	-0.29 (0.19)	-0.68 (1.23)	-0.29 (0.19)	-0.58 (1.23)
Sex (=male)	-0.05 (0.01)	-0.3 (0.07)	-0.05 (0.01)	-0.3 (0.07)
First_born	0.23 (0.01)	-0.56 (0.07)	0.27 (0.01)	-0.5 (0.07)
WIMD				
1	-0.1 (0.01)	-0.35 (0.12)	-0.07 (0.01)	-0.39 (0.11)
2	-0.04 (0.01)	-0.28 (0.12)	-0.01 (0.01)	-0.29 (0.12)
3	-0.01 (0.01)	-0.2 (0.12)	0.01 (0.01)	-0.21 (0.12)
4	0.01 (0.01)	0.01 (0.13)	0.02 (0.01)	0 (0.13)
smoking	-0.07 (0.01)	-0.46 (0.08)	NA	NA
maternal age	-0.01 (0)	-0.02 (0.01)	NA	NA
Birthweight				
CF	-96.83 (60.83)	115.32 (223.35)	-98.85 (61.72)	162.06 (221.58)
Sex (=male)	141.16 (2.83)	92.8 (14.01)	141.27 (2.87)	92.6 (14)
First_born	-102.35 (2.96)	-109.92 (14.64)	-111.49 (2.89)	-102.66 (14.09)
WIMD				
1	-52.62 (4.76)	-25.32 (23.96)	-104.03 (4.62)	-79.27 (22.85)
2	-23.76 (4.74)	-17.46 (23.86)	-60.77 (4.7)	-59.43 (23.18)
3	-8.71 (4.84)	-11.32 (24.55)	-34.06 (4.85)	-45.79 (24.1)
4	-3.44 (5.08)	-27.07 (25.77)	-18.16 (5.13)	-49.89 (25.53)
Gestational age	137.77 (1.22)	176.88 (2.57)	137.6 (1.25)	173.89 (2.6)
smoking	-154.72 (3.08)	-129 (15.02)	NA	NA
maternal age	2.56 (0.27)	3.29 (1.27)	NA	NA

Sensitivity to misclassification of CF cases

In order to assess the potential impact of misclassification of CF cases on our results, we repeated the analyses using a more stringent criterion for CF cases. In Denmark, only those individuals who had a CF code as primary or supplementary diagnosis in the Danish National Patient Register and who had been admitted to hospital more than once were classified as having CF in this sensitivity analysis. This resulted in 379 CF cases out of 1,736,783 individuals. In Wales, we only classified those individuals with CF code in the Congenital Anomaly Register and Information Service has having CF. This lead to 181 individuals with CF out of 442,664 individuals. Tables S14 and S15 below give the parameter estimates and standard errors for the sensitivity analyses as well as the original analysis (control analysis) in Denmark and Wales, respectively. Table S16 gives the estimated direct, indirect and total effects of CF in the total population in Denmark and Wales, as well as the results from the original analysis for comparison.

In Denmark the estimated parameters and effect of CF on birthweight did not differ markedly in the sensitivity compared to the original analysis. In Wales, the point estimates for the total and the direct effect increased compared to the original analysis, whereas the indirect effect decreased slightly. The confidence intervals, however, were compatible with the results from the original analysis.

Table S14: Parameter estimates (standard errors) for the model fit to the data where CF cases are identified based on CF codes in the Danish National Patient Registry and more than one hospital admission in the Danish population. The parameter estimates from the original analysis (control analysis) are also given for comparison. Values are rounded to two digits.

	Sensitivity analysis		Control analysis	
Covariate	primary component	secondary component	primary component	secondary component
Gestational age				
CF	-0.35 (0.08)	-1.18 (0.53)	-0.26 (0.06)	-1.84 (0.42)
Sex (=male)	-0.02 (0)	-0.28 (0.02)	-0.02 (0)	-0.28 (0.02)
first born	0.12(0)	-0.89 (0.02)	0.12 (0)	-0.89 (0.02)
Maternal education				
Lower secondary education, second stage of basic education	0.06 (0.01)	-0.42 (0.13)	0.06 (0.01)	-0.42 (0.13)
Upper secondary education	0.15 (0.01)	0.14 (0.13)	0.15 (0.01)	0.14 (0.13)
Post secondary non tertiary education	0.24 (0.07)	0.71 (0.72)	0.24 (0.07)	0.71 (0.72)
First stage of tertiary educ., not adv. research qualification	0.21 (0.01)	0.41 (0.13)	0.21 (0.01)	0.41 (0.13)
Second stage of tertiary educ., adv. research qualification	0.22 (0.03)	0.97 (0.29)	0.22 (0.03)	0.97 (0.29)
Not known	0.23 (0.03)	0.24 (0.33)	0.23 (0.03)	0.24 (0.33)
Birthweight				
CF	-121.95 (13.75)	-99.09 (28.99)	-107.65 (13.75)	-201.48 (29)
Sex (=male)	140.15 (25.69)	70.93 (71.48)	140.15 (20.54)	70.91 (55.48)
first born	-120.34 (0.75)	-45.07 (3.15)	-120.34 (0.75)	-45.16 (3.15)
Maternal education				
Lower secondary education, second stage of basic education	9.98 (0.82)	-94.06 (3.26)	9.92 (0.82)	-94.32 (3.27)
Upper secondary education	97.93 (4.52)	-31.89 (21.04)	97.86 (4.52)	-32.25 (21.05)
Post secondary non tertiary education	136.01 (4.5)	-117.51 (21)	135.88 (4.5)	-116.8 (21.01)
First stage of tertiary	136.85 (24.9)	-2.25 (114.15)	136.79 (24.9)	-2.64 (114.17)

educ., not adv. research qualification				
Second stage of tertiary educ., adv. research qualification	163.23 (4.52)	-62.44 (21.09)	163.17 (4.52)	-62.87 (21.09)
Not known	-34.91 (10.29)	-2.67 (46.54)	-34.96 (10.29)	-3.22 (46.56)
gestational age	137.7 (11.83)	178.81 (55)	137.72 (11.83)	178.85 (55.03)

Table S15: Parameter estimates (standard errors) for the model fit to the data where CF cases are identified based on CARIS alone in the Welsh population. The parameter estimates from the original analysis (control analysis) are also given for comparison. Values are rounded to two digits.

	Sensitivity Analysis		Control Analysis	
	primary component	secondary component	primary component	secondary component
Gestational age				
CF	-0.46 (0.11)	-0.37 (0.99)	-0.46 (0.09)	-1.47 (0.76)
Sex=male	-0.04 (0)	-0.26 (0.04)	-0.04 (0)	-0.26 (0.04)
First born	0.26 (0)	-0.58 (0.04)	0.26 (0)	-0.58 (0.04)
WIMD				
1	-0.06 (0.01)	-0.52 (0.06)	-0.06 (0.01)	-0.52 (0.06)
2	-0.01 (0.01)	-0.34 (0.06)	-0.01 (0.01)	-0.34 (0.06)
3	0 (0.01)	-0.21 (0.06)	0 (0.01)	-0.21 (0.06)
4	0.02 (0.01)	-0.04 (0.07)	0.02 (0.01)	-0.04 (0.07)
Birthweight				
CF	-170.59 (36.9)	-70.62 (189.02)	-140.68 (30.83)	-19.04 (130.97)
Sex=male	141.23 (1.48)	68.36 (7.15)	141.24 (1.48)	68.3 (7.15)
First born	-119.27 (1.5)	-82.49 (7.24)	-119.27 (1.5)	-82.48 (7.24)
WIMD				
1	-117.25 (2.37)	-91.04 (11.61)	-117.22 (2.37)	-91.09 (11.61)
2	-71.8 (2.42)	-65.41 (11.86)	-71.79 (2.42)	-65.41 (11.86)
3	-40.09 (2.48)	-36.96 (12.2)	-40.07 (2.48)	-36.95 (12.2)
4	-17.65 (2.55)	-32.13 (12.7)	-17.63 (2.55)	-32.21 (12.7)
Gestational age	135.96 (0.66)	168.78 (1.48)	135.96 (0.66)	168.75 (1.48)

Table S16: Estimated direct, indirect and total effects of CF on birthweight in the total population in Wales and Denmark using a more stringent criterion for the classification of CF cases, as well as the results from the original analysis for comparison.

	Wales		Denmark	
	Sensitivity Analysis	Control analysis	Sensitivity Analysis	Control Analysis
CF direct (95% CI)	-159.54 (-226.92 , -95.55)	-127.24 (-182 , -72.61)	-119.9 (-156.75, -71.92)	-116.09 (-152.53 , -80.5)
CF indirect (95% CI)	-62.88 (-133.8 , -17.4)	-82.85 (-122.22 , -44.91)	-63.36 (-88.64, -37.85)	-62.71 (-84.47 , -39.96)
CF total (95% CI)	-222.42 (-306.35 , -148.78)	-210.08 (-281.97 , -141.5)	-183.26 (-223.59, -113.1)	-178.8 (-225.43 , -134.47)

S.8 The probability of having low birthweight

In our fitted model the probability for a baby to be born with low birthweight depends on its covariate values, c, and the model parameters, θ. Let $F(x|c,\theta)$ denote the cumulative distribution

function of birthweight for given sets of values of c and θ. Our best guess at the probability of low birth-weight for a given set of values for c is $F(2500|c,\hat{\theta})$ where $\hat{\theta}$ is the maximum likelihood estimate of θ. To allow for the uncertainty in $\hat{\theta}$ we proceed as follows.

Draw an independent random sample of size 10 000 from the multivariate Normal sampling distribution of $\hat{\theta}$. For each sampled value, θ_i say , calculate $q_i(c) = F(2500|c,\theta_i)$ for each set of possible values of c. Our Monte Carlo estimates of the probability of low birth-weight are the sample means, q(c) say, of the $q_i(c)$. We carried out the above simulation study initially using a model which did not include an adjustment for gestational age and therefore captured the total effect of CF on birthweight. We then repeated the study using the model in the main article, which includes an adjustment for gestational age. We estimated the probabilities of low birthweight at gestational ages 35 weeks, 37 weeks and 39 weeks. This does not take into account that babies with CF are born earlier but comparing the probabilities with and without adjustment for gestational age shows that the difference between the probabilities of being born with low birthweight for CF and non-CF babies is not solely explained by differences in gestational age.

We repeated the simulation studies for all possible combinations of covariate values.

The results are given in Tables S18-S25 below.

The probability for CF babies to be born with low birthweight is between 1.3 and 1.8 and between 1.2 and 2.1 times that of non-CF babies in Wales and Denmark, respectively, and depends on sex, first-born status, deprivation and gestational age. The ratio between the probabilities for CF and non-CF babies to be born with low birthweight increases with increasing gestational age. For all of the combinations of sex, first born status and gestational age, the probability of being born with low birthweight decreases slightly with decreasing deprivation. This is the case for both, CF and non-CF babies.

Predictions in the Welsh population

Table S178: Probabilities for a baby with CF to be born with low birthweight and ratios of the probabilities of being low birthweight for CF compared to non-CF babies with the same covariate values; demographic characteristics: first born and female.

	WIMD=1		WIMD=2		WIMD=3		WIMD=4		WIMD=5	
	P(lbw)	ratio	P(lbw)	ratio	P(lbw)	ratio	P(lbw)	ratio	P(lbw)	ratio
No adjustment for gestational age	0.17 (0.14,0.19)	1.72 (1.46,1.98)	0.15 (0.12,0.17)	1.72 (1.45,2)	0.14 (0.11,0.16)	1.73 (1.45,2.01)	0.13 (0.11,0.15)	1.74 (1.44,2.03)	0.12 (0.1,0.15)	1.75 (1.45,2.05)
Born at 35 weeks	0.52 (0.47,0.57)	1.28 (1.16,1.4)	0.48 (0.43,0.54)	1.31 (1.17,1.44)	0.46 (0.41,0.51)	1.32 (1.18,1.47)	0.44 (0.39,0.49)	1.33 (1.18,1.48)	0.42 (0.37,0.47)	1.35 (1.19,1.5)
Born at 37 weeks	0.29 (0.25,0.33)	1.45 (1.24,1.67)	0.26 (0.22,0.3)	1.47 (1.25,1.71)	0.23 (0.2,0.27)	1.49 (1.26,1.74)	0.22 (0.18,0.26)	1.5 (1.26,1.76)	0.21 (0.17,0.25)	1.52 (1.27,1.79)

Born at 39 weeks	0.12 (0.1,0.15)	1.64 (1.32,2)	0.1 (0.08,0.13)	1.66 (1.32,2.04)	0.09 (0.07,0.11)	1.68 (1.32,2.07)	0.08 (0.07,0.1)	1.68 (1.32,2.09)	0.08 (0.06,0.1)	1.7 (1.32,2.12)

Table S19: Probabilities for a baby with CF to be born with low birthweight and ratios of the probability of being low birthweight for CF compared to non-CF babies with the same covariate values; demographic characteristics: not first born and female

	WIMD=1		WIMD=2		WIMD=3		WIMD=4		WIMD=5	
	P(lbw)	ratio	P(lbw)	ratio	P(lbw)	ratio	P(lbw)	ratio	P(lbw)	ratio
No adjustment for gestational age	0.13 (0.11,0.16)	1.75 (1.46,2.04)	0.12 (0.1, 0.14)	1.76 (1.44,2.06)	0.11 (0.09,0.13)	1.77 (1.43,2.1)	0.1 (0.08,0.12)	1.78 (1.43,2.13)	0.1 (0.08,0.12)	1.8 (1.43,2.16)
Born at 35 weeks	0.42 (0.37,0.47)	1.35 (1.19,1.5)	0.38 (0.34,0.43)	1.37 (1.2,1.54)	0.36 (0.31,0.4)	1.39 (1.21,1.57)	0.34 (0.3, 0.39)	1.4 (1.21,1.59)	0.32 (0.28,0.37)	1.41 (1.22,1.61)
Born at 37 weeks	0.21 (0.17,0.24)	1.52 (1.27,1.78)	0.18 (0.15,0.22)	1.54 (1.27,1.82)	0.16 (0.13,0.2)	1.55 (1.27,1.85)	0.15 (0.13,0.18)	1.56 (1.27,1.87)	0.14 (0.12,0.17)	1.57 (1.28,1.89)
Born at 39 weeks	0.08 (0.06,0.1)	1.69 (1.32,2.12)	0.06 (0.05,0.08)	1.7 (1.31,2.15)	0.06 (0.04,0.07)	1.71 (1.3, 2.18)	0.05 (0.04,0.07)	1.7 (1.29,2.18)	0.05 (0.04,0.06)	1.72 (1.3, 2.22)

Table S20: Probabilities for a baby with CF to be born with low birthweight and ratios of the probability of being low birthweight for CF compared to non-CF babies with the same covariate values; demographic characteristics: first born and male.

	WIMD=1		WIMD=2		WIMD=3		WIMD=4		WIMD=5	
	P(lbw)	ratio	P(lbw)	ratio	P(lbw)	ratio	P(lbw)	ratio	P(lbw)	ratio
No adjustment for gestational age	0.13 (0.11,0.15)	1.66 (1.39,1.92)	0.11 (0.09,0.13)	1.66 (1.37,1.93)	0.1 (0.09,0.12)	1.67 (1.36,1.95)	0.1 (0.08,0.12)	1.67 (1.35,1.97)	0.1 (0.08,0.11)	1.69 (1.35,2)
Born at 35 weeks	0.4 (0.36,0.45)	1.35 (1.19,1.52)	0.37 (0.32,0.42)	1.38 (1.2,1.56)	0.34 (0.3,0.39)	1.39 (1.21,1.58)	0.33 (0.28,0.37)	1.4 (1.22,1.6)	0.31 (0.27,0.36)	1.42 (1.22,1.62)
Born at 37 weeks	0.2 (0.16,0.23)	1.52 (1.26,1.79)	0.17 (0.14,0.2)	1.54 (1.27,1.83)	0.15 (0.13,0.19)	1.55 (1.27,1.86)	0.14 (0.12,0.17)	1.56 (1.27,1.87)	0.14 (0.11,0.16)	1.57 (1.27,1.89)
Born at 39 weeks	0.07 (0.06,0.09)	1.68 (1.3, 2.11)	0.06 (0.05,0.08)	1.68 (1.29,2.14)	0.05 (0.04,0.07)	1.69 (1.28,2.17)	0.05 (0.04,0.06)	1.68 (1.26,2.17)	0.04 (0.03,0.06)	1.69 (1.27,2.2)

Table S21: Probabilities for a baby with CF to be born with low birthweight and ratios of the probability of being low birthweight for CF compared to non-CF babies with the same covariate values; demographic characteristics: not first born and male

	WIMD=1		WIMD=2		WIMD=3		WIMD=4		WIMD=5	
	P(lbw)	ratio	P(lbw)	ratio	P(lbw)	ratio	P(lbw)	ratio	P(lbw)	ratio
No adjustment for gestational age	0.1 (0.08,0.12)	1.69 (1.37,1.98)	0.09 (0.07,0.11)	1.69 (1.35,2.02)	0.09(0.07, 0.1)	1.71 (1.33,2.06)	0.08 (0.06,0.1)	1.72 (1.32,2.1)	0.08 (0.06,0.1)	1.74 (1.32,2.14)
Born at 35 weeks	0.31 (0.27,0.35)	1.42 (1.22,1.62)	0.28 (0.24,0.32)	1.44 (1.23,1.65)	0.25 (0.22,0.3)	1.45 (1.23,1.68)	0.24 (0.2,0.28)	1.46 (1.23,1.69)	0.23 (0.19,0.27)	1.47 (1.24,1.71)
Born at 37 weeks	0.13 (0.11,0.16)	1.57 (1.27,1.89)	0.12 (0.09,0.14)	1.58 (1.26,1.92)	0.1 (0.08,0.13)	1.58 (1.26,1.94)	0.1 (0.08,0.12)	1.58 (1.25,1.95)	0.09 (0.07,0.11)	1.59 (1.25,1.97)
Born at 39 weeks	0.04 (0.03,0.06)	1.68 (1.26,2.19)	0.04 (0.03,0.05)	1.67 (1.22,2.21)	0.03 (0.02,0.04)	1.66 (1.2,2.23)	0.03 (0.02,0.04)	1.64 (1.17,2.24)	0.03 (0.02,0.04)	1.66 (1.17,2.27)

Predictions in the Danish population

Table S22: Probabilities for a baby with CF to be born with low birthweight and ratios of the probability of being low birthweight for CF compared to non-CF babies with the same covariate values; demographic characteristics: first born and female

	Education level 1		Education level 2		Education level 3		Education level 4		Education level 5		Education level 6		Education level 7	
	P(lbw)	ratio	P(lbw)	ratio	P(lbw)	ratio	P(lbw)	ratio	P(lbw)	ratio	P(lbw)	ratio	P(lbw)	ratio
No adjustment for gestational age	0.13 (0.12, 0.15)	1.57 (1.42, 1.72)	0.13 (0.12, 0.14)	1.52 (1.39, 1.66)	0.11 (0.1, 0.12)	1.57 (1.43, 1.72)	0.1 (0.08, 0.11)	1.58 (1.37, 1.85)	0.1 (0.09, 0.1)	1.6 (1.45, 1. 75)	0.09 (0.08, 0.1)	1.64 (1.47, 1.83)	0.13 (0.12, 0.15)	1.58 (1.43, 1.76)
Born at 35 weeks	0.5 (0.47, 0.53)	1.25 (1.17, 1.33)	0.5 (0.47, 0.53)	1.25 (1.16, 1.33)	0.42 (0.39, 0.46)	1.29 (1.19, 1.38)	0.4 (0.35, 0.45)	1.29 (1.19, 1.4)	0.39 (0.36, 0.42)	1.31 (1.2,1.41)	0.38 (0.34, 0.41)	1.31 (1.2,1.42)	0.53 (0.49, 0.57)	1.24 (1.16, 1.31)
Born at 37 weeks	0.27 (0.24, 0.3)	1.41 (1.27, 1.56)	0.27 (0.24, 0.3)	1.41 (1.27, 1.55)	0.21 (0.19, 0.24)	1.47 (1.31, 1.63)	0.2 (0.17, 0.23)	1.47 (1.31, 1.64)	0.19 (0.17, 0.21)	1.49 (1.33, 1.67)	0.18 (0.16, 0.21)	1.5 (1.33, 1.67)	0.29 (0.26, 0.32)	1.39 (1.26, 1.53)
Born at 39 weeks	0.11 (0.09, 0.12)	1.62 (1.4,1.86)	0.11 (0.09, 0.12)	1.63 (1.41, 1.86)	0.08 (0.07, 0.09)	1.71 (1.46, 1.98)	0.07 (0.06, 0.1)	1.72 (1.47, 2.01)	0.07 (0.06, 0.08)	1.75 (1.48, 2.03)	0.06 (0.05, 0.08)	1.76 (1.5,2.05)	0.12 (0.1,0.14)	1.59 (1.38, 1.82)

Table S23: Probabilities for a baby with CF to be born with low birthweight and ratios of the probability of being low birthweight for CF compared to non-CF babies with the same covariate values; demographic characteristics: not first born and female

	Education level 1		Education level 2		Education level 3		Education level 4		Education level 5		Education level 6		Education level 7	
	P(lbw)	ratio	P(lbw)	ratio	P(lbw)	ratio	P(lbw)	ratio	P(lbw)	ratio	P(lbw)	ratio	P(lbw)	ratio
No adjustment for gestational age	0.1 (0.09, 0.12)	1.64 (1.48, 1.81)	0.1 (0.1, 0.11)	1.58 (1.44, 1.73)	0.08 (0.08, 0.09)	1.65 (1.48, 1.82)	0.08 (0.06, 0.09)	1.67 (1.41, 2.01)	0.08 (0.07, 0.08)	1.7 (1.51, 1.89)	0.07 (0.06, 0.08)	1.75 (1.53, 1.99)	0.1 (0.09, 0.12)	1.66 (1.48, 1.86)
Born at 35 weeks	0.4 (0.37, 0.43)	1.3 (1.2, 1.41)	0.4 (0.37, 0.43)	1.3 (1.2, 1.4)	0.33 (0.3, 0.36)	1.34 (1.23, 1.46)	0.31 (0.27, 0.35)	1.34 (1.22, 1.47)	0.3 (0.28, 0.33)	1.36 (1.24, 1.49)	0.29 (0.26, 0.32)	1.36 (1.24, 1.49)	0.43 (0.39, 0.47)	1.29 (1.19, 1.39)
Born at 37 weeks	0.19 (0.17, 0.22)	1.49 (1.32, 1.67)	0.2 (0.18, 0.22)	1.48 (1.32, 1.65)	0.15 (0.13, 0.17)	1.54 (1.36, 1.74)	0.14 (0.12, 0.17)	1.54 (1.35, 1.75)	0.13 (0.12, 0.15)	1.57 (1.38, 1.78)	0.13 (0.11, 0.15)	1.57 (1.38, 1.78)	0.21 (0.19, 0.24)	1.47 (1.31, 1.63)
Born at 39 weeks	0.07 (0.06, 0.08)	1.73 (1.47, 2.02)	0.07 (0.06, 0.08)	1.74 (1.48, 2.01)	0.05 (0.04, 0.06)	1.83 (1.54, 2.15)	0.05 (0.04, 0.07)	1.83 (1.52, 2.18)	0.04 (0.04, 0.05)	1.87 (1.56, 2.22)	0.04 (0.03, 0.05)	1.88 (1.57, 2.24)	0.08 (0.07, 0.09)	1.7 (1.45, 1.97)

Table S24: Probabilities for a baby with CF to be born with low birthweight and ratios of the probability of being low birthweight for CF compared to non-CF babies with the same covariate values; demographic characteristics: first born and male.

	Education level 1		Education level 2		Education level 3		Education level 4		Education level 5		Education level 6		Education level 7	
	P(lbw)	ratio	P(lbw)	ratio	P(lbw)	ratio	P(lbw)	ratio	P(lbw)	ratio	P(lbw)	ratio	P(lbw)	ratio
No adjustment for gestational age	0.1 (0.09, 0.11)	1.57 (1.42, 1.72)	0.1 (0.09, 0.11)	1.51 (1.38, 1.64)	0.08 (0.08, 0.09)	1.57 (1.42, 1.71)	0.08 (0.06, 0.09)	1.58 (1.35, 1.89)	0.08 (0.07, 0.08)	1.61 (1.45, 1.76)	0.07 (0.06, 0.08)	1.65 (1.46, 1.86)	0.1 (0.09, 0.11)	1.59 (1.43, 1.77)
Born at 35 weeks	0.39 (0.35, 0.42)	1.32 (1.21, 1.42)	0.38 (0.35, 0.42)	1.31 (1.2, 1.41)	0.32 (0.29, 0.35)	1.35 (1.23, 1.48)	0.3 (0.26, 0.34)	1.35 (1.23, 1.49)	0.29 (0.26, 0.32)	1.37 (1.25, 1.51)	0.28 (0.25, 0.31)	1.37 (1.25, 1.51)	0.41 (0.38, 0.45)	1.3 (1.2, 1.4)
Born at 37 weeks	0.18 (0.16, 0.21)	1.51 (1.33, 1.69)	0.19 (0.16, 0.21)	1.5 (1.33, 1.67)	0.14 (0.12, 0.16)	1.56 (1.37, 1.76)	0.13 (0.11, 0.16)	1.55 (1.36, 1.77)	0.12 (0.11, 0.14)	1.59 (1.39, 1.81)	0.12 (0.1, 0.14)	1.59 (1.39, 1.8)	0.2 (0.18, 0.23)	1.48 (1.32, 1.66)
Born at 39 weeks	0.06 (0.05, 0.07)	1.75 (1.48, 2.05)	0.07 (0.06, 0.08)	1.76 (1.49, 2.05)	0.05 (0.04, 0.05)	1.85 (1.55, 2.19)	0.05 (0.03, 0.06)	1.86 (1.54, 2.22)	0.04 (0.03, 0.05)	1.9 (1.58, 2.26)	0.04 (0.03, 0.05)	1.91 (1.58, 2.28)	0.07 (0.06, 0.09)	1.71 (1.46, 1.99)

Table S25: Probabilities for a baby with CF to be born with low birthweight and ratios of the probability of being low birthweight for CF compared to non-CF babies with the same covariate values; demographic characteristics: not first born and male.

	Education level 1		Education level 2		Education level 3		Education level 4		Education level 5		Education level 6		Education level 7	
	P(lbw)	ratio	P(lbw)	ratio	P(lbw)	ratio	P(lbw)	ratio	P(lbw)	ratio	P(lbw)	ratio	P(lbw)	ratio
No adjustment for gestational age	0.08 (0.07, 0.09)	1.65 (1.48, 1.82)	0.08 (0.08, 0.09)	1.58 (1.43, 1.72)	0.07 (0.06, 0.08)	1.67 (1.48, 1.85)	0.06 (0.05, 0.08)	1.69 (1.4,2.09)	0.06 (0.06, 0.07)	1.72 (1.51, 1.93)	0.06 (0.05, 0.07)	1.79 (1.53, 2.06)	0.08 (0.07, 0.09)	1.68 (1.49, 1.89)
Born at 35 weeks	0.3 (0.27, 0.33)	1.38 (1.25, 1.51)	0.3 (0.27, 0.33)	1.36 (1.24, 1.49)	0.24 (0.22, 0.26)	1.41 (1.27, 1.56)	0.22 (0.19, 0.26)	1.4 (1.26, 1.56)	0.22 (0.19, 0.24)	1.43 (1.29, 1.59)	0.21 (0.18, 0.23)	1.43 (1.29, 1.58)	0.32 (0.29, 0.35)	1.36 (1.24, 1.48)
Born at 37 weeks	0.13 (0.11, 0.14)	1.59(1.39,1.81)	0.13 (0.11, 0.15)	1.58 (1.38, 1.78)	0.1 (0.08, 0.11)	1.65 (1.43, 1.88)	0.09 (0.07, 0.12)	1.62 (1.4,1.89)	0.09 (0.07, 0.1)	1.68 (1.45, 1.93)	0.08 (0.07, 0.1)	1.67 (1.44, 1.92)	0.14 (0.12, 0.16)	1.57 (1.37, 1.77)
Born at 39 weeks	0.04 (0.03, 0.05)	1.88 (1.56, 2.23)	0.04 (0.03, 0.05)	1.88 (1.57, 2.23)	0.03 (0.02, 0.03)	1.99 (1.62, 2.41)	0.03 (0.02, 0.04)	1.98 (1.57, 2.43)	0.02 (0.02, 0.03)	2.05 (1.65, 2.5)	0.03 (0.02, 0.03)	2.05 (1.63, 2.52)	0.04 (0.04, 0.05)	1.84 (1.54, 2.17)

References

1. VanderWeele TJ, Vansteelandt S: **Conceptual issues concerning mediation, interventions and composition**. *Statistics and Its Interface* 2009, **2**:457-468.
2. VanderWeele TJ: **Mediation Analysis: A Practitioner's Guide**. *Annu Rev Public Health* 2016, **37**:17-32.
